# Supplementary material for: Employing automatic content recognition for teaching methodology analysis in classroom videos
Source: PLoS One. 2022 Feb 17;17(2):e0263448. doi: 10.1371/journal.pone.0263448 (PMC8853489; doi:10.1371/journal.pone.0263448)
Supplement: Attachment — Submitted filename: Response to Reviewers.pdf [file pone.0263448.s002.pdf]

Revision Response Note [PONE-D-21-18820]
Employing Automatic Content Recognition for Teaching Methodology Analysis in Classroom Videos

We are grateful to the respected editor, the editorial team and erudite reviewers for extending this opportunity to improve our manuscript. We are thankful for the insightful comments and valuable suggestions by the erudite reviewers. It encouraged us to improve the proposed study and the quality of the manuscript. We diligently deliberated the suggestions, supplemented the proposed study and ameliorated the manuscript accordingly. We address the journal requirements guidelines and comments of each respected reviewer separately in the following:

Journal Requirements:
When submitting your revision, we need you to address these additional requirements.
1. Please ensure that your manuscript meets PLOS ONE's style requirements, including those for file naming. The PLOS ONE style templates can be found at https://journals.plos.org/plosone/s/file?id=wjVg/PLOSOne_formatting_sample_main_body.pdf and https://journals.plos.org/plosone/s/file?id=ba62/PLOSOne_formatting_sample_title_authors_affiliations.pdf

Response: *The revised manuscript complies the PLOS ONE's style requirements.*

2. Please note that PLOS ONE does not allow footnotes, so please include all text in the footnotes in the main text. Please also include the name of the Ethics committee which approved the study, and clarify how participants provided consent.

Response: *The footnotes are accommodated in the text and a letter including the names of members of ethics committee with details about consent is provided as supplementary document.*

3. Thank you for stating the following in the Acknowledgments Section of your manuscript:
"We would like to thank the University of Management and Technology for providing the 430 CCTV camera lecture videos. This work was supported by a grant from the Institute of 431 Information & Communications Technology Planning & Evaluation (IITP) funded by 432 the Korean government (MSIT) (No. 2014-3-00077, AI National Strategy Project)."
We note that you have provided additional information within the Acknowledgements Section that is not currently declared in your Funding Statement. Please note that funding information should not appear in the Acknowledgments section or other areas of your manuscript. We will only publish funding information present in the Funding Statement section of the online submission form.
Please remove any funding-related text from the manuscript and let us know how you would like to update your Funding Statement. Currently, your Funding Statement reads as follows:
"This work was supported by Institute of Information & communications Technology Planning & Evaluation (IITP) grant funded by the Korea government (MSIT) (No.2014-3-00077, AI National Strategy Project)."
Please include your amended statements within your cover letter; we will change the online submission form on your behalf.

Response: *A revised funding and acknowledgment statement is provided in cover letter.*

4. We note that Figure 1 includes an image of a [patient / participant / in the study]. As per the PLOS ONE policy (http://journals.plos.org/plosone/s/submission-guidelines#loc-human-subjects-research) on papers that include identifying, or potentially identifying, information, the individual(s) or parent(s)/guardian(s) must be informed of the terms of the PLOS open-access (CC-BY) license and provide specific permission for publication of these details under the terms of this license. Please download the Consent Form for Publication in a PLOS Journal (http://journals.plos.org/plosone/s/file?id=8ce6/plos-consent-form-english.pdf). The signed consent form should not be submitted with the manuscript, but should be securely filed in the individual's case notes. Please amend the methods section and ethics statement of the manuscript to explicitly state that the patient/participant has provided consent for publication: “The individual in this manuscript has given written informed consent (as outlined in PLOS consent form) to publish these case details”.
If you are unable to obtain consent from the subject of the photograph, you will need to remove the figure and any other textual identifying information or case descriptions for this individual.

Response: *A letter from the ethics committee providing the consent of the participants is provided as a supplementary document.*

5. Please include captions for your Supporting Information files at the end of your manuscript, and update any in-text citations to match accordingly. Please see our Supporting Information guidelines for more information: http://journals.plos.org/plosone/s/supporting-information.

Response: *The requirement is accommodated in the revised manuscript.*

Additional Editor Comments (if provided):
The authors have to address the comments of the reviewers.
[Note: HTML markup is below. Please do not edit.]
Reviewers' comments:
Reviewer's Responses to Questions

Comments to the Author

1. Is the manuscript technically sound, and do the data support the conclusions?

The manuscript must describe a technically sound piece of scientific research with data that supports the conclusions. Experiments must have been conducted rigorously, with appropriate controls, replication, and sample sizes. The conclusions must be drawn appropriately based on the data presented.
Reviewer #1: Partly
Reviewer #2: Yes

Response: *We are grateful for the acknowledgment by the reviewers and improved the deficiencies with the help of suggested changes and feedback.*

2. Has the statistical analysis been performed appropriately and rigorously?
Reviewer #1: Yes
Reviewer #2: No

Response: *We acknowledge the insightful feedback provided by the reviewers and revised the manuscript anticipating the astute observations by the reviewers.*

---

3. Have the authors made all data underlying the findings in their manuscript fully available?

The PLOS Data policy requires authors to make all data underlying the findings described in their manuscript fully available without restriction, with rare exception (please refer to the Data Availability Statement in the manuscript PDF file). The data should be provided as part of the manuscript or its supporting information, or deposited to a public repository. For example, in addition to summary statistics, the data points behind means, medians and variance measures should be available. If there are restrictions on publicly sharing data—e.g. participant privacy or use of data from a third party—those must be specified.

Reviewer #1: No
Reviewer #2: Yes

Response: *We are grateful for the acknowledgment by the reviewers.*

---

4. Is the manuscript presented in an intelligible fashion and written in standard English?

PLOS ONE does not copyedit accepted manuscripts, so the language in submitted articles must be clear, correct, and unambiguous. Any typographical or grammatical errors should be corrected at revision, so please note any specific errors here.

Reviewer #1: Yes
Reviewer #2: Yes

Response: *We are grateful for the acknowledgment by the reviewers.*

---

5. Review Comments to the Author

Please use the space provided to explain your answers to the questions above. You may also include additional comments for the author, including concerns about dual publication, research ethics, or publication ethics. (Please upload your review as an attachment if it exceeds 20,000 characters)

Reviewer #1: The article presents the importance of the existence of a module for recognizing human actions that can be used to improve the teaching act. Also, this includes a description of how the dataset was collected and three types of video classifiers.
I suggest the following improvements:

Section 2.1 should detail the preprocessing stage (what transforms are used for data preprocessing before training). It is specified that augmentation was used only in the results section.

Response: *Section 2.1 is updated with the pre-processing and data augmentation information. Moreover, the details are appended in section 3 (Results, Discussion and limitation). Briefly, this study uses horizontal flip, zoom-in, zoom-out, change of brightness, image rotation and image blur for data augmentation.*

Figure 2 can be improved because it is not explicit enough.
Response: *Figure 2 has been updated in the manuscript and the caption provides additional information to remove the ambiguities. The description below is also updated section 2 of the manuscript for better readability and understanding.*

- it is not clear if the first architecture uses only the first and last image or the whole video;
  Response: *It takes one image at one time step 't' and predicts an action after t_N (N number of images).*
- in the second architecture, it is not clear if only three frames;
  Response: *It takes one image at one time step 't' and predicts an action after t_N (N number of images). However, in implementation we can pass all N images at the same time.*
- it is not clear how the prediction of the action is determined, starting from the output of the LSTMs;
  Response: *The action is predicted at the end of a sequence.*
- it is not clear whether a convolutional layer is used for each image or all images are passed through the same layer.
  Response: *CNN is used for each image in architectures 1 and 2. While in architecture 3 all the images are clamped at once to the 3DCNN.*

Section 3 contains general information about various types of neural networks but does not include a clear presentation of the proposed methods and the pipeline used to train them. This section could be renamed and used as Background and introduced a new section with the presentation of the proposed methods (containing a presentation specific to the problem solved).

Response: *As per the suggestion, a new subsection has been added to the manuscript with precise details of the proposed techniques relevant to the selected problem.*

In section 4, it is not specified how the ADAM optimizer was chosen, what value was used for the learning rate and if other variants were tested.
Response: *The details for Adam optimizer have been added in section 3 (Results, Discussion and Limitations).*

Figure 6 could also include the percentages for each type of action. The size used for the batch is not specified. The error function used is not specified.
Response: *As per suggestion Figure 6 is updated and the details of batch size and the error /loss function information are added in section 3 (Results, Discussion and Limitations).*

It would be useful if Table 2 turned into a figure. It would be easier to understand the proposed model.
Response: *As per suggestion Table 1 and Table 2 are transformed into figures (Figure 10 (a) and (b)).*

The authors should point out the contributions they bring in the context of human activity recognition or video classification compared to the other approaches presented in the state-of-the-art section.
Response: *The introduction section has been updated with elaborated contributions.*

A comparison should be made between the results obtained by these three methods presented.
Response: *The comparison is presented in discussion section and updated in Table 3.*

Reviewer #2: Comments to the Author:
This paper has proposed a teaching methodology analysis in classroom videos. The work of this paper is practical. However, I think there are some revisions for the manuscript. The comments are list as follow.

(1) The author needs to analyze the difference of effect for each label.
Response: It is added to the section 2.1 of the manuscript.

(2) The author does not reflect the novelty of the method. The method used is the existing method. Whether it is advanced in this field needs to be compared with the existing advanced method in this field.
Response: *The introduction section is updated with elaborated contributions and novelty of the idea in this study.*

(3) The latest references cited by the author contain too few articles in this direction.
Response: *The reference section is updated with recent relevant articles.*

(4) The first appearance abbreviation should show all words, such as CCTV in line 71 and IDTs in line 77.
Response: *The manuscript is carefully revised and all words are used for the first use of an abbreviation.*

---

6. PLOS authors have the option to publish the peer review history of their article (what does this mean?). If published, this will include your full peer review and any attached files.

If you choose “no”, your identity will remain anonymous but your review may still be made public.

Do you want your identity to be public for this peer review? For information about this choice, including consent withdrawal, please see our Privacy Policy.

Reviewer #1: No
Reviewer #2: No

[NOTE: If reviewer comments were submitted as an attachment file, they will be attached to this email and accessible via the submission site. Please log into your account, locate the manuscript record, and check for the action link "View Attachments". If this link does not appear, there are no attachment files.]

While revising your submission, please upload your figure files to the Preflight Analysis and Conversion Engine (PACE) digital diagnostic tool, https://pacev2.apexcovantage.com/. PACE helps ensure that figures meet PLOS requirements. To use PACE, you must first register as a user. Registration is free. Then, login and navigate to the UPLOAD tab, where you will find detailed instructions on how to use the tool. If you encounter any issues or have any questions when using PACE, please email PLOS at figures@plos.org. Please note that Supporting Information files do not need this step.

Response: *All figures are adjusted according to the instructions provided by PACE.*
