# Supplementary material for: Employing automatic content recognition for teaching methodology analysis in classroom videos
Source: PLoS One. 2022 Feb 17;17(2):e0263448. doi: 10.1371/journal.pone.0263448 (PMC8853489; doi:10.1371/journal.pone.0263448)
Supplement: Attachment — Submitted filename: Revision_Response_2.pdf [file pone.0263448.s003.pdf]

Revision Response Note [PONE-D-21-18820R1]
Employing Automatic Content Recognition for Teaching Methodology Analysis in Classroom Videos

We are grateful to the respected editor and the editorial team. We are also grateful for the insightful comments and valuable suggestions by the erudite reviewers. The feedback encouraged us to improve the proposed study and the quality of the manuscript. We diligently deliberated the suggestions and ameliorated the manuscript accordingly. We address the comments of the knowledgeable reviewers in the following sections:

Journal Requirements:
Please review your reference list to ensure that it is complete and correct. If you have cited papers that have been retracted, please include the rationale for doing so in the manuscript text, or remove these references and replace them with relevant current references. Any changes to the reference list should be mentioned in the rebuttal letter that accompanies your revised manuscript. If you need to cite a retracted article, indicate the article's retracted status in the References list and also include a citation and full reference for the retraction notice.

Response: *We have tried our best to ensure completeness and correctness of the references list. The revised manuscript complies with the PLOS ONE's style requirements.*

Additional Editor Comments (if provided):
The authors have to address the comments of the reviewers.
[Note: HTML markup is below. Please do not edit.]
Reviewers' comments:
Reviewer's Responses to Questions

Comments to the Author

1. If the authors have adequately addressed your comments raised in a previous round of review and you feel that this manuscript is now acceptable for publication, you may indicate that here to bypass the "Comments to the Author" section, enter your conflict of interest statement in the "Confidential to Editor" section, and submit your "Accept" recommendation.
Reviewer #1: (No Response)

Reviewer #2: All comments have been addressed

Response: *We are grateful for the acknowledgment by the reviewers and improved the deficiencies with the help of suggested changes and feedback.*

---

2. Is the manuscript technically sound, and do the data support the conclusions?
The manuscript must describe a technically sound piece of scientific research with data that supports the conclusions. Experiments must have been conducted rigorously, with appropriate controls, replication, and sample sizes. The conclusions must be drawn appropriately based on the data presented.

Reviewer #1: Partly
Reviewer #2: Yes
Response: *We acknowledge the insightful feedback provided by the reviewers and revised the manuscript anticipating the astute observations by the reviewers.*

---

3. Has the statistical analysis been performed appropriately and rigorously?
Reviewer #1: Yes
Reviewer #2: Yes
Response: *We appreciate the positive feedback provided by the reviewers.*

---

4. Have the authors made all data underlying the findings in their manuscript fully available?

The PLOS Data policy requires authors to make all data underlying the findings described in their manuscript fully available without restriction, with rare exception (please refer to the Data Availability Statement in the manuscript PDF file). The data should be provided as part of the manuscript or its supporting information, or deposited to a public repository. For example, in addition to summary statistics, the data points behind means, medians and variance measures should be available. If there are restrictions on publicly sharing data—e.g. participant privacy or use of data from a third party—those must be specified.

Reviewer #1: No
Reviewer #2: Yes

Response: *We are grateful for the acknowledgment by the reviewers.*

---

5. Is the manuscript presented in an intelligible fashion and written in standard English?

PLOS ONE does not copyedit accepted manuscripts, so the language in submitted articles must be clear, correct, and unambiguous. Any typographical or grammatical errors should be corrected at revision, so please note any specific errors here.

Reviewer #1: Yes
Reviewer #2: Yes

Response: *We are grateful for the acknowledgment by the reviewers.*

---

6. Review Comments to the Author

Please use the space provided to explain your answers to the questions above. You may also include additional comments for the author, including concerns about dual publication, research ethics, or publication ethics. (Please upload your review as an attachment if it exceeds 20,000 characters)

Reviewer #1: Preprocessing information has been added, but it is too minimal. It was not specified with what probability these transformations were applied and what parameters are used for each transform (i.e. the scale used for zooming in / zooming out).
Response : *As suggested by the reviewer the details of data augmentation operations used in this study are elaborated with selected parameters in section 3 (line 409-420 - page 22) ("Results, Discussion and Limitations").*

The authors did not specify all parameters for the neural networks (ConvLSTM / 3DCNN). For example, no parameters were specified for LSTMs such as the number of features in the hidden state h, the number of recurrent layers. The two proposed models should be better detailed. Some of this information existed in the previous table.
Response : *As suggested by the reviewer the details from the mentioned tables are added to the images. The hyper-parameters of the neural networks are added in the text in images and elaborated in the captions. The details are pronounced in the text added earlier for clarity and better readability. (Figure 10 (image and caption), Section 2.3.1, line 359-362, page 22)*

The authors provide general details: "The input of Conv3D is five-dimensional; the 1st dimension is the number of samples, the 2nd dimension is depth, the 3rd is width, the 4th is height, and the 5th is the number of channels. In the video dataset, samples are numbers of videos, and depth is the number of frames in a video". What is the significance of the number of videos in the context of the proposed solution?
Response : *The notified sentence is rephrased to clearly express the authors intention. The video clips (batch size) often play a critical role as an implicit regularizer, which in this study can not have much impact as a single clip is used in a batch. The frames in a clip form a volume and are assumed to serve the purpose of the batch, but the claims need further inquiry which can be pursued in future work.*

The authors provided details for the optimization algorithm using notation symbols. It is necessary to explain what each notation represents. For example, alpha represents the learning rate, epsilon represents the term added to the denominator to improve numerical stability.

Response : *As suggested by the reviewer the descriptions are added to the manuscript in the Section 3 (line 393-395 – page 22) " Results, Discussion and Limitations". Moreover, following reference is added for a detailed reading.*

"Kingma DP, Ba J. Adam: A Method for Stochastic Optimization. In: 3rd International Conference on Learning Representations, ICLR , San Diego, CA, USA, May 7-9, 2015."

Reviewer #2: Comments to the Author:
The author has made great modification to the structure of the paper. Recent references have been added as required. Meanwhile, the relative methods are compared. The paper has met the basic requirements of the journal. I don't have any problems. I suggest to accept it.
Response : *We are grateful to the respected reviewer for providing us with valuable comments which helped us to improve the manuscript and prepare it for positive appraisal.*

7. PLOS authors have the option to publish the peer review history of their article (what does this mean?). If published, this will include your full peer review and any attached files.

If you choose “no”, your identity will remain anonymous but your review may still be made public.

Do you want your identity to be public for this peer review? For information about this choice, including consent withdrawal, please see our Privacy Policy.

Reviewer #1: No
Reviewer #2: No

[NOTE: If reviewer comments were submitted as an attachment file, they will be attached to this email and accessible via the submission site. Please log into your account, locate the manuscript record, and check for the action link "View Attachments". If this link does not appear, there are no attachment files.]

While revising your submission, please upload your figure files to the Preflight Analysis and Conversion Engine (PACE) digital diagnostic tool, https://pacev2.apexcovantage.com/. PACE helps ensure that figures meet PLOS requirements. To use PACE, you must first register as a user. Registration is free. Then, login and navigate to the UPLOAD tab, where you will find detailed instructions on how to use the tool. If you encounter any issues or have any questions when using PACE, please email PLOS at figures@plos.org. Please note that Supporting Information files do not need this step.

Response: *All figures are adjusted according to the instructions provided by PACE.*
