# Supplementary material for: Employing automatic content recognition for teaching methodology analysis in classroom videos
Source: PLoS One. 2022 Feb 17;17(2):e0263448. doi: 10.1371/journal.pone.0263448 (PMC8853489; doi:10.1371/journal.pone.0263448)
Supplement: Attachment — Submitted filename: Review for PONE-D-21-18820.docx [file pone.0263448.s001.docx]

**Comments to the Author:**

**This paper has proposed a teaching methodology analysis in classroom videos. The work of this paper is practical. However, I think there are some revisions for the manuscript. The comments are list as follow.**

**(1) The author needs to analyze the difference of effect for each label.**

**(2) The author does not reflect the novelty of the method. The method used is the existing method. Whether it is advanced in this field needs to be compared with the existing advanced method in this field.**

**(3) The latest references cited by the author contain too few articles in this direction.**

**(4) The first appearance abbreviation should show all words, such as CCTV in line 71 and IDTs in line 77.**
